# Current AI Applications and Challenges in Oral Pathology

**DOI:** 10.3390/oral5010002

**Published:** 2025-01-06

**Authors:** Zaizhen Xu, Alice Lin, Xiaoyuan Han

**Affiliations:** Department of Biomedical Sciences, Arthur A. Dugoni School of Dentistry, University of the Pacific, 155 5th St., San Francisco, CA 94103, USA

**Keywords:** artificial intelligence, oral pathology, oral cancer, machine learning, deep learning

## Abstract

Artificial intelligence (AI), particularly through machine learning (ML) and deep learning (DL) techniques such as convolutional neural networks (CNNs) and natural language processing (NLP), has shown remarkable promise in image analysis and clinical documentation in oral pathology. In order to explore the transformative potential of artificial intelligence (AI) in oral pathology, this review highlights key studies demonstrating current AI’s improvement in oral pathology, such as detecting oral diseases accurately and streamlining diagnostic processes. However, several limitations, such as data quality, generalizability, legal and ethical considerations, financial constraints, and the need for paradigm shifts in practice, are critically examined. Addressing these challenges through collaborative efforts, robust validation, and strategic integration can pave the way for AI to revolutionize oral pathology, ultimately improving patient outcomes and advancing the field.

## 1. Introduction

Artificial intelligence (AI) is a branch of computer science dedicated to creating machines capable of performing tasks that typically require human intelligence. Within AI, machine learning (ML) is a pivotal subset, enabling machines to learn patterns directly from data without explicit programming. Deep learning (DL) represents an advanced subset of ML, characterized by training neural networks with many layers, hence the term “deep”. An artificial neural network (ANN) is a core structure in DL, utilizing interconnected units called neurons to solve complex tasks by learning from data. A specialized form of ANN, the convolutional neural network (CNN), excels at capturing the spatial hierarchy of data. In CNNs, early layers detect simple features like edges and colors, and deeper layers identify complex features such as shapes in images. The architecture of CNNs makes them ideal for image recognition and analysis.

Oral pathologists face significant challenges, including the time-consuming nature of manually analyzing large volumes of histopathological images, the risk of human error in detecting subtle abnormalities, and the variability in diagnostic interpretations between pathologists. Both ML and DL have demonstrated significant advancements in early detection, diagnosis, and prognostic prediction, which aids medical decision-making for both specialists and patients [1]. In dentistry, the use of ML has been demonstrated to effectively improve decision-making by identifying key factors in orthodontic procedures and evaluating the longevity of restorative materials [2,3]. DL, because of its ability to perform complex visual analyses, underscores its potential in enhancing diagnostic accuracy and clinical workflows in oral pathology [4].

We searched the keywords of “artificial intelligence”, “oral pathology”, “deep learning”, and/or “oral cancer” in PubMed and Google Scholar between 2020 to 2024. Here, we reviewed the current AI applications and challenges in oral pathology. Understanding the application and addressing the limitations of AI use in oral pathology will improve its effectiveness and patient outcomes.

## 2. Current Studies on AI’s Application in Diagnosis of Oral Cancer

This section reviewed current studies focusing on the use of AI, specifically CNNs, for diagnosing oral cancer and related conditions. AI has been applied in the detection of other oral diseases including dental caries [5], osteonecrosis of the jaw [6], orthodontic treatment [7], etc. Here, we focus on AI use in oral cancer diagnosis (Table 1).

### 2.1. Applications in Intraoral Photography for Oral Cancer Detection

The study conducted by Wang, Liu, and Wu presents a significant advancement in the application of AI for the early diagnosis of oral cancer, utilizing clinical images taken by regular cameras. The proposed method combines a deep belief network (DBN) with a combined group teaching optimization (CGTO) algorithm to enhance the precision and efficiency of diagnostic processes. This hybrid approach involves initial preprocessing steps, such as noise reduction and contrast enhancement, which improve the quality of the raw image data. The DBN is then optimized using the CGTO algorithm, leading to superior diagnostic performance. The study achieved remarkable results, with a precision rate of 97.71%, a sensitivity rate of 92.37%, a Matthews correlation coefficient of 94.65%, and an F1 score of 94.65% [8]. These metrics indicate the method’s high accuracy in distinguishing between cancerous and non-cancerous samples. The advantage of this methodology lies in its ability to integrate deep learning and metaheuristic optimization, providing a robust and efficient diagnostic tool that outperforms traditional techniques like ANN, Bayesian, and CNN models. However, the study also faces challenges such as the small size of the dataset and potential class imbalance, which could impact the generalizability of the results. Despite these limitations, the DBN-CGTO method represents a promising step forward in leveraging AI for clinical applications in oral pathology, potentially leading to improved early detection and patient outcomes.

### 2.2. Applications in Pathological Slides for Oral Cancer Diagnosis

Whole slide images (WSIs) are digitalized high-resolution images of the entire histological slides, which avoid bias due to image capture and expand the analyzed data to the whole biopsy. Using WSIs, the study by Ahmed et al. introduces a hybrid diagnostic system using CNN fusion features for early detection of oral squamous cell carcinoma (OSCC). The methodology involves enhancing image quality, segmenting regions of interest with the adaptive region growing algorithm, and extracting features using CNN models (GoogLeNet, ResNet101, VGG16). These features are then fused and classified using neural networks, including ANN, and XGBoost, which is a combination of multiple weak models (typically decision trees) that together form a strong model. The results demonstrate high diagnostic accuracy, with the fused CNN features yielding an AUC of 98.85%, accuracy of 99.3%, sensitivity of 98.2%, precision of 99.5%, and specificity of 98.35% [9]. The advantage of this approach is the significant reduction of human error, providing consistent and reliable results, and improved early detection of OSCC. However, the study also highlights several disadvantages. The reliance on high-quality, balanced datasets is critical; limited or biased data can compromise the model’s performance and generalizability. The interpretability of deep learning models remains a challenge, as they often function as black boxes, making it difficult to understand the decision-making process. This lack of transparency can hinder the acceptance and trust of AI-based diagnoses among healthcare professionals and patients.

### 2.3. Applications in Noninvasive Screening for Oral Cancer

The examination of the stain slides under the microscope is the gold standard in pathology diagnosis, but this method involves invasive painful biopsies, complex slide preparation, and the necessity of skilled pathologists for interpretation. Sunny et al. offered a minimally invasive alternative with AI prediction that can rapidly process and analyze samples at the point of care, potentially leading to quicker clinical decisions and interventions [10]. This platform, known as Cellscope, is a portable, automated system that digitizes cytology slides and utilizes a trained ANN to classify atypical cells and stratify risks associated with oral potentially malignant (OPML) and malignant lesions. The study conducted a thorough evaluation involving 82 subjects diagnosed with OPML or malignant lesions, demonstrating a notable overall accuracy of 84–86% in detecting oral lesions. It also reported that the tele-cytology platform achieved a good agreement with conventional direct microscopy (Kappa value 0.67–0.72), further validating its efficacy as a complementary tool in the diagnostic workflow. One of the primary advantages of this tele-cytology platform is its portability and automation, which makes it especially suited for deployment in low-resource settings where access to specialized pathology services is limited. The platform allows frontline health workers to capture high-resolution cytology images and transmit them to remote experts for analysis, facilitating timely and accurate diagnosis. Furthermore, the ANN’s ability to automate the classification of cells reduces the subjectivity and variability inherent in manual cytological assessments, enhancing consistency and reliability in diagnostic outcomes. Despite these advantages, there are some notable disadvantages. The study highlighted that the platform’s sensitivity in detecting high-grade dysplasia (HGD), an oral lesion with a high risk of malignant transformation, was relatively low at 18%, which is a limitation shared with conventional cytology. This reduced sensitivity can be attributed to the challenges of obtaining cells from deeper layers of highly keratinized stratified squamous epithelium, an issue that both manual and automated methods currently face. Additionally, while the ANN improved detection accuracy, its training and validation require substantial computational resources and a large dataset of high-quality annotated images, which may not always be available in all settings.

Optical coherence tomography (OCT) provides high-resolution, cross-sectional images of tissues of living patients by utilizing a handheld device that directs light into the tissue and captures the reflected light in a painless manner. Yuan et al. investigated the use of a novel deep learning method, the local residual adaptation network (LRAN), for noninvasive oral cancer screening using OCT images [11]. The LRAN is specifically designed to address the challenges associated with OCT-based cancer detection by integrating two key components: the residual feature representation (RFR) module and the local distribution adaptation (LDA) module. The RFR module is used to extract high-level feature representations from the OCT images. This approach helps capture intricate details in the images that are crucial for distinguishing between cancerous and non-cancerous tissues. The LDA module further enhances the model’s performance by minimizing the distribution discrepancies between the training and testing datasets, allowing the model to adapt to variations in the imaging conditions and still perform accurately during real-world applications. This dual-module approach allows the LRAN to achieve high diagnostic performance, with an accuracy of 91.62%, sensitivity of 91.66%, and specificity of 92.58% on a dataset of 26,400 2D images derived from 264 3D OCT images collected from 25 patients [11]. The advantages of this method include the noninvasive nature of OCT, which avoids the need for painful biopsies, and its ability to deliver high diagnostic accuracy, potentially improving early detection and patient outcomes in oral cancer. However, the study also highlights some limitations, such as the need for large-scale, high-quality image datasets to effectively train the model, and the challenges associated with adapting the model to different imaging conditions, which could affect its generalizability. The future implications of this research are significant, as it demonstrates the potential for more accurate, efficient, and patient-friendly cancer screening methods, with possible applications extending to other types of malignancies.

## 3. Current Studies on AI’s Application in Enhancing Workflow

The integration of AI into oral pathology workflows has shown significant potential to enhance diagnostic efficiency and accuracy. This section reviews AI tools used in oral pathology, including Impetus, QuPath, and NLP in report management. These advancements highlight AI’s transformative impact on oral pathology, aiming to improve diagnostic precision and streamline clinical processes.

### 3.1. Impetus

The study by Gu et al. introduced Impetus, an AI tool for assisting pathologists in tumor detection from histological slides. The tool employs CNN feature extraction. The AI categorizes slides into high, medium, and low confidence levels. In high-confidence cases, the AI classifies slides, and pathologists confirm or override the diagnosis. In medium-confidence scenarios, pathologists provide a differential diagnosis, while low-confidence cases require full manual examination. The tool was tested with eight pathologists using lymph node slides for detecting breast cancer metastasis. Results showed that while the AI could efficiently highlight areas of interest, the detection of small lesions remained a challenge, critical for early diagnosis. Pathologists often supplemented the AI’s annotations, particularly in low-confidence cases, where they performed manual diagnoses, reflecting a cautious approach to AI reliance. The study also emphasized the importance of incorporating patient medical history and using implicit feedback from pathologists, such as areas of focus and zoom levels, to improve the AI’s accuracy and usability [23]. Although the tool showed promise in enhancing diagnostic workflows, the pathologists’ low trust in fully automated diagnosis highlighted the need for ongoing human oversight. Future improvements could focus on integrating comprehensive clinical data to enhance AI decision-making reliability.

### 3.2. QuPath

The study by Bankhead et al. introduces QuPath, an open-source software for digital pathology and WSI analysis. The study involved using QuPath to analyze immunohistochemistry markers such as CD3, CD8, p53, and PD-L1 in a cohort of colorectal cancer patients. The software’s features include annotation, viewing, and interactive machine learning-based classification, with integration capabilities for tools like ImageJ. Clinically, QuPath could standardize and accelerate biomarker analysis, enhancing diagnostic accuracy and patient stratification. However, limitations include the technical expertise required for customization and challenges in handling diverse staining patterns, which may limit its broader clinical adoption. The study concludes that while QuPath offers significant research and clinical potential, further development and validation are necessary to ensure seamless integration into clinical workflows [24]. QuPath’s utility in standardizing research and reducing bias was further demonstrated in a study by Moratin et al. [25], which applied digital pathology algorithms to analyze the expression levels of biomarkers like PD-L1, EGFR, and COX-2 in a large cohort of oral cancers. The study found that digital scoring of QuPath provided reliable and objective quantification, correlating well with manual scoring and allowing for the identification of prognostic markers. This digital approach helps mitigate observer bias, enhancing the reproducibility and accuracy of biomarker research. However, challenges such as intratumor heterogeneity and the need for comprehensive validation in diverse clinical settings remain. These findings underscore the potential of digital pathology tools like QuPath to revolutionize biomarker research and clinical diagnostics by providing more consistent and objective data analysis [25].

### 3.3. Natural Language Processing (NLP) in Report Management

Natural language processing (NLP) is a field of artificial intelligence that focuses on the interaction between computers and humans through natural language, enabling machines to understand, interpret, and respond to human language effectively [26,27].

One application of NLP is an AI scribe, which is an automated system designed to assist with transcription, documentation, and writing tasks by accurately converting spoken language into written text. The benefit of incorporating AI scribes, as demonstrated by the one provided by Permanente Medical Group, includes significant time savings for physicians, reducing documentation burdens by an average of one hour per day [28]. Using a secure smartphone, the ambient AI scribe transcribes patient encounters in real time and uses ML to produce clinical notes, filtering out non-clinical conversations to create precise documentation. The AI scribe’s ability to accurately and efficiently document clinical encounters improves workflow and accuracy, which can be particularly beneficial in oral pathology, as it allows oral pathologists to describe their findings verbally while examining slides, reducing the time wasted by switching between looking at slides and typing. This also minimizes the need for correcting typing errors, streamlining the diagnostic process and improving accuracy.

In the study by Zhang et al., an NLP algorithm was developed to transcribe oral examination data into a structured dental charting database. The study involved using case vignettes for primary, mixed, and permanent dentition patients with varying degrees of soft tissue pathology, caries, existing restorations, and occlusion relationships. Dental students conducted simulated oral exams, and the algorithm, implemented in JAVA, was calibrated and validated against human charting performance. Results showed the algorithm achieved a recall rate of 99.0% and a precision rate of 97.8%, comparable to human accuracy [29]. However, while the tool is highly accurate, there are occasional instances of “hallucinations”, where the AI incorrectly interprets the clinical content, highlighting the need for ongoing refinement and validation.

NLP can also be applied to enhance the management and analysis of oral pathology reports, which are typically written in free text. By converting these reports into structured data, NLP can assist in identifying specific oral pathologies, categorizing stages of diseases, and tracking treatment outcomes over time. The study by Yim et al. demonstrates methods for converting unstructured clinical text into structured data using algorithms for text processing and relation extraction. It identified 7112 unique patients with new pulmonary nodules, achieving an 87% positive predictive value and 96% negative predictive value compared with clinician review of the medical records [30]. This demonstrates the potential of NLP in facilitating better integration of oral pathology data with other medical records, providing a more comprehensive view of a patient’s health and aiding in multidisciplinary care approaches. As in oncology, the application of NLP in oral pathology could reduce the need for labor-intensive manual data extraction, enhance the quality of research by providing access to a wealth of previously untapped data, and ultimately lead to improved patient outcomes. However, limitations exist, such as the dependency on the quality and consistency of the input data, the need for extensive annotated corpora for training the systems, and the variability in performance across different medical domains and institutions.

## 4. Challenges of AI in Oral Pathology

While the application of AI has the potential to significantly aid pathologists in the diagnosis of oral diseases and enhance workflow efficiency, it is critical to acknowledge its limitations and define clear boundaries for its use. Recognizing the limitations of AI and establishing clear boundaries for its application are essential. Pathologists must be equipped to critically evaluate AI-generated results, mitigating the risk of errors stemming from over-reliance or misuse. Addressing these challenges and fostering a collaborative approach between AI and human expertise will unlock the full potential of AI in oral pathology, paving the way for more accurate diagnoses and improved patient care.

### 4.1. Training Data Quality and Size

One of the primary limitations of AI models in oral pathology is the quality and size of the training data sets. Large high-quality datasets are essential for training robust AI models. However, variability in tissue fixation, cutting, and staining procedures can lead to inconsistencies that impact the performance of AI models. AI systems trained on flawed or biased data may produce unreliable results, and detecting these flaws may not be immediate. Furthermore, once flawed data are distributed, they can persist on the web for years, continuing to affect AI models even after the original source is retracted [31]. The challenge of small datasets is particularly acute in specialized areas of oral pathology where rare conditions may not have enough documented cases to form a robust training set. A potential solution includes training AI models with larger, more comprehensive datasets and performing local calibration to account for variations. Multi-institutional collaborations can help pool data, enhancing the overall quality and diversity of training sets [32].

### 4.2. Generalization

Generalization is another major limitation. AI models in oral pathology often struggle to generalize across different patient cohorts and clinical settings. These models are typically designed for specific tasks and may not perform well when applied broadly due to differences in patient populations and clinical environments. The specificity of AI models means they excel in certain tasks but can degrade when exposed to data from different populations or settings not represented in the training data. Differences in patient demographics, disease prevalence, and clinical practices across regions can destabilize the accuracy of AI models, making them less reliable in diverse settings. Improving generalization requires training AI models on diverse datasets that include a wide range of patient demographics and clinical scenarios. Continuous updating and local calibration of models are essential to maintain accuracy and reliability [32].

### 4.3. Legal and Ethical Concerns

The use of AI in oral pathology raises several legal and ethical issues that must be addressed to ensure compliance and trust. Ensuring that AI systems comply with patient privacy regulations, such as HIPAA in the United States, is crucial. The use of patient data for training and validation must be carefully managed to protect confidentiality. Additionally, AI tools must undergo rigorous validation and approval processes to meet regulatory standards. This can be a lengthy and complex process, hindering the rapid adoption of new AI technologies [31]. Legal issues also encompass the need for compliance with regional and international standards, which can vary significantly. This compliance ensures that the AI models used are not only effective but also ethically sound, maintaining the trust of both patients and healthcare providers.

### 4.4. Financial Constraints

Implementing AI systems in oral pathology is financially demanding. The costs associated with setting up the necessary infrastructure, including hardware and software, maintaining and updating systems, and pushing products to commercialization are substantial. Many companies struggle to commercialize their AI methods, resulting in several “orphan” products that are approved but not widely used in practice. Financial constraints also include the ongoing costs for system updates and training of personnel to use these advanced systems effectively.

### 4.5. Paradigm Shift in Practice

The integration of AI into oral pathology requires a significant shift in the current paradigms of practice. There is often a lack of trust in AI systems, with many pathologists unfamiliar with their operation and benefits. Historical parallels, such as the resistance to microscopy in the 19th century, highlight the challenges of adopting new technologies [33]. Current workflows in pathology are often inexpensive and effective, based primarily on microscope-based diagnoses. Transitioning to AI-based systems requires not only technical upgrades but also a shift in mindset among pathologists, who must learn to trust and effectively use these new tools. The success of this transition varies significantly across medical fields, partly due to differences in working styles and the nature of tasks performed [32].

Thus, while AI holds great potential for advancing oral pathology, significant limitations related to data quality, generalization, legal and financial constraints, and the need for a paradigm shift among pathologists must be addressed. Overcoming these challenges will require collaborative efforts and robust strategies to ensure the successful integration of AI into clinical practice.

## 5. Future Perspective

Moreover, advancements in AI-driven noninvasive diagnostic techniques, such as optical coherence tomography (OCT) and tele-cytology platforms, are expected to improve accessibility to high-quality pathology services in resource-limited settings. These technologies can expand the reach of oral pathology, enabling early detection and intervention in underserved regions.

Finally, addressing ethical and regulatory considerations will remain central to AI’s future in oral pathology. Establishing frameworks to ensure patient privacy, data security, and compliance with international standards will be crucial for the widespread adoption and acceptance of AI systems. With continued innovation, strategic integration, and interdisciplinary collaboration, AI holds the potential to revolutionize oral pathology, enhance diagnostic accuracy, and improve patient outcomes on a global scale.

## 6. Conclusions

Research on AI models demonstrated AI’s capabilities in detecting oral pathologies, assisting diagnostic decision-making, and improving clinical workflows. However, challenges such as data variability, limited training datasets, and generalizability issues must be addressed. Ensuring patient privacy, regulatory compliance, and managing financial constraints are also critical. Additionally, successful integration requires education and a mindset shift among pathologists. While AI has the potential to revolutionize oral pathology, overcoming these hurdles through collaboration, validation, and strategic implementation is key to enhancing diagnostics and improving patient outcomes.

## Figures and Tables

**Table 1.** Current AI application in Diagnosis of Oral Cancer.

Image Type	AI Model	Model Performance	Advantage	Limitation	Publication	Other Reports
Clinical photo images	Deep belief network with combined group teaching optimization	Precision: 97.71%; Sensitivity: 92.37%	High precision and efficiency	Small dataset, class imbalance	[8]	[9–12]
Whole slide images	CNN fusion features with ANN and XGBoost	Accuracy: 99.3%; Precision: 99.5%; Sensitivity: 98.2%; Specificity: 98.35%	Reduces human error, improves early detection	High-quality datasets needed, black box nature of deep learning	[13]	[14–16]
Cytology slides	Automated system using ANN	Accuracy: 84–86%	Portability, automation, suited for low-resource settings	Low sensitivity for high-grade dysplasia, requires large datasets	[17]	[18,19]
Optical coherence tomography images	Local residual adaptation network	Accuracy: 91.62%; Sensitivity: 91.66%; Specificity: 92.58%	Noninvasive, high diagnostic accuracy	Large datasets required, variations in imaging conditions	[20]	[21,22]

Abbreviation notes: CNN—convolutional neural network; ANN—artificial neural network.
